# Characterization of a novel reassortment Tibet orbivirus isolated from *Culicoides* spp. in Yunnan, PR China

**DOI:** 10.1099/jgv.0.001645

**Published:** 2021-09-08

**Authors:** Nanjie Ren, Xiaoyu Wang, Mengying Liang, Shen Tian, Christabel Ochieng, Lu Zhao, Doudou Huang, Qianfeng Xia, Zhiming Yuan, Han Xia

**Affiliations:** ^1^​ Key Laboratory of Special Pathogens and Biosafety, Wuhan Institute of Virology, Chinese Academy of Sciences, Wuhan, Hubei, PR China; ^2^​ University of Chinese Academy of Sciences, Beijing, PR China; ^3^​ Key Laboratory of Tropical Translational Medicine of Ministry of Education, NHC Key Laboratory of Control of Tropical diseases,School of Tropical Medicine and Laboratory Medicine, Hainan Medical University, Haikou, PR China

**Keywords:** *Culicoides*, reassortment, Tibet orbivirus, Yunnan

## Abstract

Orbiviruses are arboviruses with 10 double-stranded linear RNA segments, and some have been identified as pathogens of dramatic epizootics in both wild and domestic ruminants. Tibet orbivirus (TIBOV) is a new orbivirus isolated from hematophagous insects in recent decades, and, currently, most of the strains have been isolated from insects in PR China, except for two from Japan. In this study, we isolated a novel reassortment TIBOV strain, YN15-283-01, from *Culicoides* spp. To identify and understand more characteristics of YN15-283-01, electrophoresis profiles of the viral genome, electron microscopic observations, plaque assays, growth curves in various cell lines, and bioinformatic analysis were conducted. The results indicated that YN15-283-01 replicated efficiently in mosquito cells, rodent cells and several primate cells. Furthermore, the maximum likelihood phylogenetic trees and simplot analysis of the 10 segments indicated that YN15-283-01 is a natural reassortment isolate that had emerged mainly from XZ0906 and SX-2017a.

## Introduction

Emerging and re-emerging arbovirus diseases remain one of the most serious threats to human and animal health, with potentially devastating social and economic consequences. For example, Zika virus (ZIKV) was unexpectedly associated with severe neurological disease (Guillain–Barré syndrome) in adults in French Polynesia [1], followed by clusters of microcephaly in Brazil [2], leading to the World Health Organization (WHO) declaring a public health emergency of international concern (PHEIC) in 2016. Dengue virus (DENV) is estimated to infect 390 million people annually, of which 96 million manifest clinically (with any severity of disease) [3]. Despite a risk of infection existing in 129 countries [4], 70% of the actual burden of DENV is in Asia. Furthermore, the emergence of the epidemic and epizootic West Nile virus (WNV) in the Americas (1999–2004), Rift Valley fever (RVF) in Africa (2009–2011) and the epidemics of chikungunya virus (CHIKV) (2004–2014) were truly black swan events (i.e., epidemics that are difficult to predict and that have an extreme effect), which had substantial public health and economic effects that took the world by surprise [5]. Since One Health is receiving attention for arbovirus infection prevention and control [6], scientists realized that globalization [7], climate change [8], increasing vector distribution [9] and urbanization [10] are all the reasons for rising incidence and geographical expansion of arboviruses.

Orbiviruses consist of 10 segments of linear double-stranded RNA and are members of the *Reoviridae* family with 14 other genera. Currently in the *Orbivirus* genus, there are 22 recognized species and at least 160 different serotypes worldwide [11]. Orbiviruses are distributed globally [12], and the majority are habitually found in tropical or subtropical zones, including Europe, Asia and Africa [13]. For a while, most attention has been given to four representatives of the genus *Orbivirus* that are known to cause economic burden and significant diseases of farm animals, bluetongue virus (BTV), epizootic haemorrhagic disease virus (EHDV) [14], African horse sickness virus (AHSV) [15] and equine encephalosis virus (EEV) [16].

Orbiviruses are transmitted between their vertebrate hosts, including domestic and wild ruminants [17], by a variety of vectors, such as mosquitoes, midges and ticks [18]. Among these vectors, members of the genus *Culicoides* [19] carry most orbiviruses and are considered a potent vector of important animal arbovirus diseases, which cause major economic losses in domestic animals [20, 21].

The first identified Tibet orbivirus (TIBOV, XZ0906) was isolated from *Anopheles maculatus* mosquito specimens collected in 2009 from Tibet, PR China. Molecular genetic analysis revealed that the isolate was a novel species within the genus *Orbivirus* [22]. Subsequently, several TIBOV strains were found in *Culex fatigan* mosquitoes and in *Culicoides* collected from Guangdong and Yunnan provinces (PR China) [23]. Additionally, almost all the TIBOVs have been found in PR China, except for two found in Japan [24]. Furthermore, serological evidence of TIBOV infection in livestock confirmed the long-term prevalence in the southwest border area of Yunnan, PR China, and that they potentially caused livestock disease outbreaks [25]. Due to the segmented structure of the virus, when coinfection occurs, genetic reassortment or exchange of segments between orbivirus species could transpire, which inevitably affects the host range, virulence, immune evasion and evolution of antiviral resistance [26–28].

In recent years, next-generation sequencing (NGS) and viral metagenomics have greatly increased the pace of new virus discovery from arthropod vectors such as mosquitoes, midges, ticks and sandflies, allowing nucleotide sequences, taxonomic assignments and phylogenetic and evolutionary relationships to be obtained without actual live virus isolates and with minimal biological data [29–31].

In this study, a Tibet orbivirus strain YN15-283-01 was isolated from specimens of members of the genus *Culicoides* collected in Yunnan in 2015, and genome sequencing revealed that the isolate is a natural reassortment virus. Cell lines derived from mosquitoes, humans, monkeys and hamsters are highly susceptible to infection by this isolate. Our findings expand the knowledge of diversity, evolutionary relationships and the characteristics of TIBOV.

## Methods

### *Culicoides* collection and sample preparation

A total of 7700 *Culicoides* were trapped in Lincang, Pu’er, Xishuangbanna and Honghe, Yunnan Province, PR China, between August and September 2015 and then assigned to 77 pools (100 midges per pool) based on their respective collection sites. Each *Culicoides* pool was triturated by the cryogenic grinding method at liquid nitrogen temperatures using sterile mortars and pestles. After sufficient grinding, 1 ml of Roswell Park Memorial Institute (RPMI) medium was added for homogenization [25], the samples were then clarified by centrifugation at 20000 ***g*** (4 °C for 30 min), filtered through a 0.22 µm membrane filter (Millipore) to remove cell debris and stored at −80 °C until further use.

### Cell culture, virus isolation and Purification

In this study, invertebrate cell lines [*Aedes albopictus* RNAi-deficient (C6/36), *Aedes aegypti* (Aag2)] and vertebrate cell lines [African green monkey kidney (Vero E6), baby hamster kidney (BHK-21), Madin-Darby bovine kidney (MDBK), human hepatoma (Huh7) and human adrenal gland (SW13)] were used.

C6/36 cells were maintained at 28 °C (in the absence of CO2) in RPMI medium (Gibco) supplemented with 10% foetal bovine serum (FBS; Gibco) and 1% penicillin/streptomycin. Aag2 cells were passaged in Schneider’s *Drosophila* Medium (SDM; Gibco) containing 5% FBS, cultured at 28 °C in an incubator with 5% CO2. Vero E6, BHK-21, SW13 and MDBK cells were grown at 37 °C under a 5% CO2 atmosphere in Dulbecco’s minimal essential medium (DMEM; Gibco) (4.5 g l^−1^ d-glucose) supplemented with 10% FBS and 1% penicillin/streptomycin.

For virus isolation, the microfiltered supernatants (200 µl) from each pool of homogenized midges were inoculated into each well of 24-well plates with C6/36 cells as passage one. After 1 h of incubation at 28 °C, the inoculum was removed and replaced with RPMI (with 2% FBS) medium, and the cell plates were put into the incubator under 28 °C with 5 % CO2 for 5–7 days, followed by two passages in C6/36 cells. Then the supernatants (200 µl) from the third passage in C6/36 were seeded on BHK-21 cells plates (24-well plates) and incubated for 5–7 days to monitor the cytopathic effects (CPEs) associated with infection.

Virus purification was performed as described previously [32, 33]. Briefly, the supernatants from viral culture were harvested and clarified to remove cellular debris by centrifugation at 5000 ***g*** at 4°C for 30 min. Thereafter, ultracentrifugation was conducted by adding 2 ml of the harvested virus supernatant at the bottom of the ultracentrifuge tube (Type 70), followed by the careful addition of 4 ml of 20% (w/v) sucrose in phosphate-buffered saline (PBS) into the tube to remove impurities. Ultracentrifugation was performed at 40000 ***g*** at 4°C for 3.5 h in a Type 70 Ti rotor (Beckman Coulter Diagnostics). Upon completion, the supernatant was discarded. Then, 100 µl of PBS was added to the sediment at the bottom of the tube and the precipitate was resuspended. The purified virus was stored at −80°C.

### Electron microscopy

Electron microscopy imaging was performed using the negative contrast method. A 20 µl sample of purified virus and an equal volume of PBS were mixed and then placed on a Formvar carbon-coated copper grid for 3 min. After staining with 2% phosphotungstic acid solution (pH 6.8) for 3 min, the excess liquid was absorbed and discarded. Finally, clear and well-contrasted electron microscope images were taken using a U8010 electron microscope (Hitachi).

### dsRNA-polyacrylamide gel electrophoresis

Viral RNA was extracted from the culture supernatant of CPE-positive BHK-21 cells using a Direct-zol RNA MiniPrep kit (Zymo Research) according to the manufacturer’s requirements. The dsRNA was unpacked in a 65 °C -water bath for 10 min and then mixed with 5 µl of 6× loading buffer. The dsRNA was then separated on a standard discontinuous 10 % acrylamide slab gel, and electrophoresis was performed in an ice bath at 80 V for 30 min, followed by 100 V for 7 h. The virus dsRNA was visualized with silver nitrate staining.

### Plaque assay

The virus was diluted 10-fold with DMEM to 10^−9^, and 100 µl virus diluent was added to the BHK-21, Vero E6 and SW13 cell monolayers in 24-well plates at 37 °C. After 1 h, the virus diluent was discarded, and 500 µl DMEM containing 1.5% methyl cellulose was added to the cells and the cells were cultured at 37 °C. Four days later, the cells were fixed overnight with 3.7% formaldehyde and stained with 2% crystal violet. The number of plaques was calculated, and the sizes of the plaques were measured.

### RNA extraction and RT-qPCR

Viral RNA was extracted from the cell culture supernatant using a Direct-zol RNA MiniPrep kit (Zymo Research) according to the manufacturer’s instructions. RT-qPCR for the detection of viral RNA was performed using a Luna Universal Probe One-Step RT-qPCR Kit (New England Biolabs) according to the manufacturer’s recommendations with a thermocycler (BIO-RAD CFX96 Real-Time System).

The primers for RT-qPCR targeted the VP1 (Segment 1) of TIBOV, including TBV-VP1-F (5′–ATCACAATGGTCGTAATAAC −3′), TBV-VP1-R (5′–TCATCATTAACTGCTAATCTTG−3′) and TBV-VP1-Probe (5′FAM–CAGATCTAATAAGACGAACAAT-BHQ13′) [34]. Both oligoprimer DNAs were synthesized by TSINGKE (Wuhan Branch, PR China). Reaction mixtures (20 µl) containing 2 µl of viral RNA and 0.8 µl of each primer were incubated at 55 °C for 10 min and 95 °C for 1 min followed by 40 cycles of 95 °C for 10 s and 55 °C for 30 s.

Standard curve generation was initiated by a 10-fold serial dilution (from 2.7×10^7^ p.f.u. ml^−1^ to 2.7×10^1^ p.f.u. ml^−1^) from viral stock, and then RNA was extracted from 200 µl of each dilution and used as the template for RT-qPCR. Each assay was carried out in triplicate. The viral titres (p.f.u. ml^−1^) were automatically plotted against cycle threshold (Ct) values based on linear regression.

### Growth characteristics of the viral isolate in the cell cultures

BHK-21, Vero E6, SW13, C6/36 and Aag2 cells grown in T25 flask plates were infected with the virus at MOI=1, 0.01 and 0.0001. Huh7 and MDBK cells grown in T25 flask plates were infected with the virus at MOI=10, 5, 1, 0.01 and 0.0001. After inoculation, 200 µl cell supernatant was collected every day (from day 0 to day 7) and then replenished with 200 µl fresh medium. Viral RNA was extracted and detected by RT-qPCR, and the virus concentration (p.f.u. ml^−1^) was calculated based on the standard curve according to the generated Ct values. The experiment was repeated three times.

### Genome sequencing and PCR confirmation

Viral RNA was extracted from the passaged virus and sent to Nextomics Biosciences (Wuhan, PR China) for sequencing through the Illumina MiSeq System. The *de novo* assembly of viral sequences was performed using MEGAHIT (https://github.com/voutcn/megahit) and then further verified with PCR using multiple primers.

### Bioinformatics analyses

The complete coding sequences (CDSs) of all ten segments of all seven isolates of TIBOV were used in the analysis (GenBank as of the first of June 2021). Background information of all the utilized strains is listed in Table 1. The nucleotide sequence identities of the CDSs of TIBOV strain YN15-283-01 and others were analysed using BioEdit v7.2.6. Alignment was conducted by the ClustalW function in MEGAv7.0.212. The GTR+I+G substitution model was selected as the best-fit nucleotide substitution model by jModelTest 23. Phylogenetic analyses of nucleotide sequences were performed by the maximum likelihood method using the general time-reversible model with 1000 bootstrap replicates in mega.

**Table 1.** Background information on viruses used in bioinformatic analysis

Viruses		Strains						
		YN15-283-01	XZ0906	SX-2017a	DH13C120	D181/2008	KSB-8/C/09	KSB-8/C/10
**Location**		Yunnan, PR China	Tibet, PR China	Yunnan, PR China	Yunnan, PR China	Guangdong, PR China	Kagoshima, Japan	Kagoshima, Japan
**Isolated source**		*Culicoides* midges	*Anopheles maculatus*	*Culex tritaeniorhynchus*	*Culicoides* midges	*Culex pipiens fatigans*	*Culicoides* midges	*Culicoides* midges
**Collection Date**		2015	2009	2007	2013	2008	2009	2010
**Accession number**	**Segment 1**	MT793636	KF746187	NC_033782	KU754026	NC_027803	LC567112	LC567102
	**Segment 2**	MT793637	KF746188	NC_033783	KU754027	NC_027811	LC567113	LC567103
	**Segment 3**	MT793638	KF746189	NC_033784	KU754028	NC_027812	LC567114	LC567104
	**Segment 4**	MT793639	KF746190	NC_033785	KU754029	NC_027813	LC567115	LC567105
	**Segment 5**	MT793640	KF746191	NC_033786	KU754030	NC_027804	LC567116	LC567106
	**Segment 6**	MT793641	KF746192	NC_033787	KU754031	NC_027814	LC567117	LC567107
	**Segment 7**	MT793642	KF746193	NC_033788	KU754032	NC_027805	LC567118	LC567108
	**Segment 8**	MT793643	KF746194	NC_033789	KU754033	NC_027806	LC567119	LC567109
	**Segment 9**	MT793644	KF746195	NC_033790	KU754034	NC_027815	LC567120	LC567110
	**Segment 10**	MT793645	KF746196	NC_033791	KU754035	NC_027807	LC567121	LC567111

Potential reassortment or recombination events in the complete genome of strain YN15-283-01 were identified using the Recombination Detection Programme (RDP 4 Version 4.33) through different algorithms (RDP, GENECONV, Bootscan, MaxChi, Chimaera, SiScan and 3Seq) [35]. The potential reassortment or recombination events were further verified by similarity plots (SimPlots) analysis in SimPlot version 3.5.1 [36].

### Statistical analyses

All statistical data were analysed with R (version 4.0.5). One-way ANOVA was used to determine significant differences among groups of highest viral titres in different cell lines under the same MOI (MOI=1, 0.01 or 0.0001) and different MOIs of the same cell line (confidence interval 95%).

## Results

### Virus isolation and morphology

The supernatants from each pool of homogenized *Culicoides* were inoculated into C6/36 cells for three times and then into BHK-21 cells. After 120 h of infection, strong CPEs in BHK-21 cells were observed, with cells becoming round and dead under the microscope. The virus isolate was obtained from one of 77 pools and named as YN15-283-01.

**Fig. 1.**
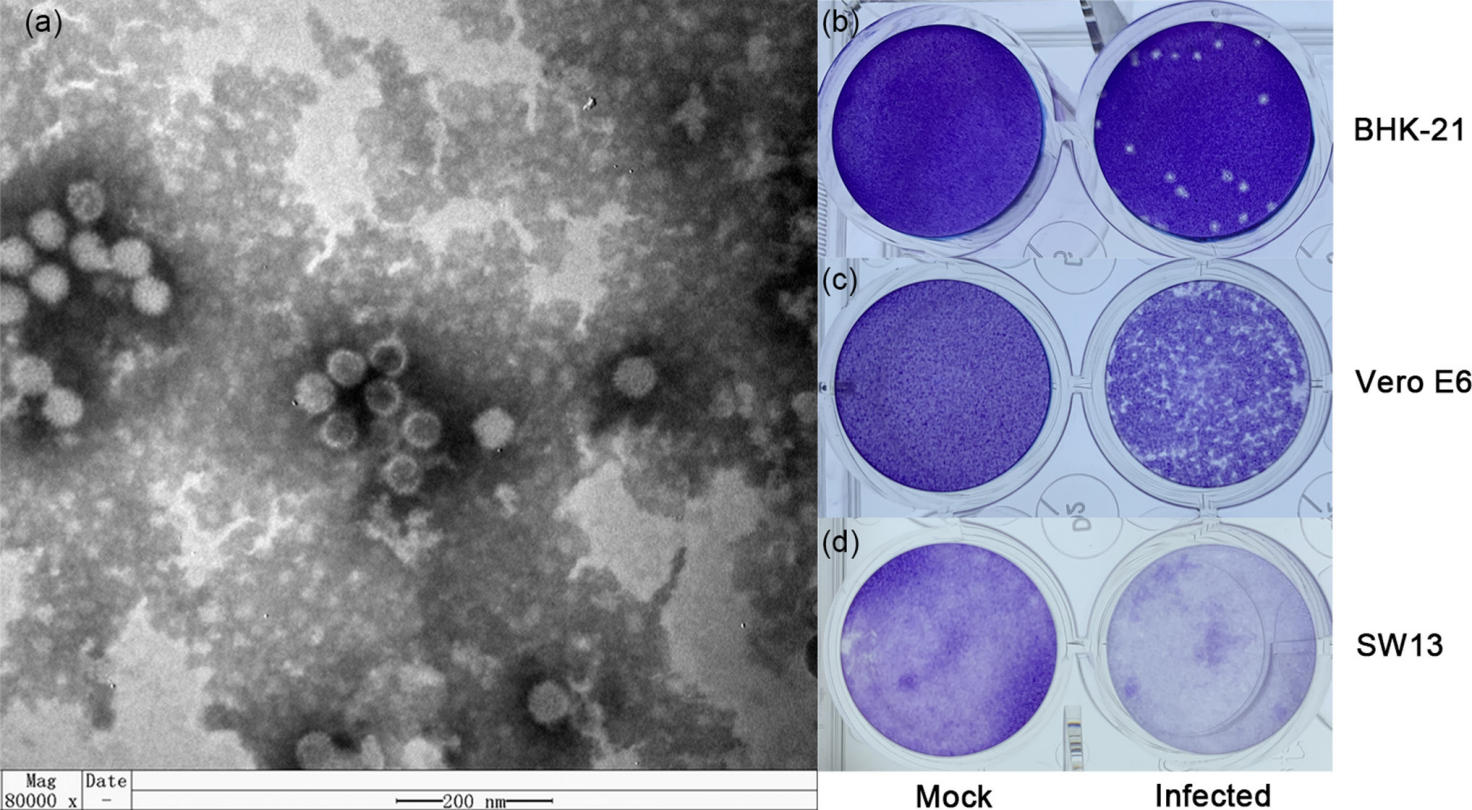
The viral morphology and viral plaques in cells of YN15-283-01. (**a**) Negative-stained ultracentrifuged virions. Viral plaques in (**b**) BHK-21 (10^−5^ dilution at four dpi), (**c**) Vero E6 (10^−3^ dilution at five dpi) and (**d**) SW13 (10^−3^ dilution at five dpi) cell monolayers. The wells were 16 mm in diameter.

Viral purification was performed by sucrose density gradient centrifugation. Electron microscopic observation revealed that the virus particle showed a spherical morphology without an envelope and a diameter of 40–60 nm (Fig. 1a).

In addition, in the plaque morphology test, YN15-283-01caused distinct, encircled plaques in BHK-21 cells (10^−5^ dilution) at day four after inoculation (Fig. 1b). However, no sharp plaques were produced (Fig. 1c) in Vero E6 cells (10^−3^ dilution) at day five after inoculation, and only detachment of the cell monolayer was observed in SW13 cells (10^−3^ dilution) on day five post-infection.

### Identification of the dsRNA genome structure

**Fig. 2.**
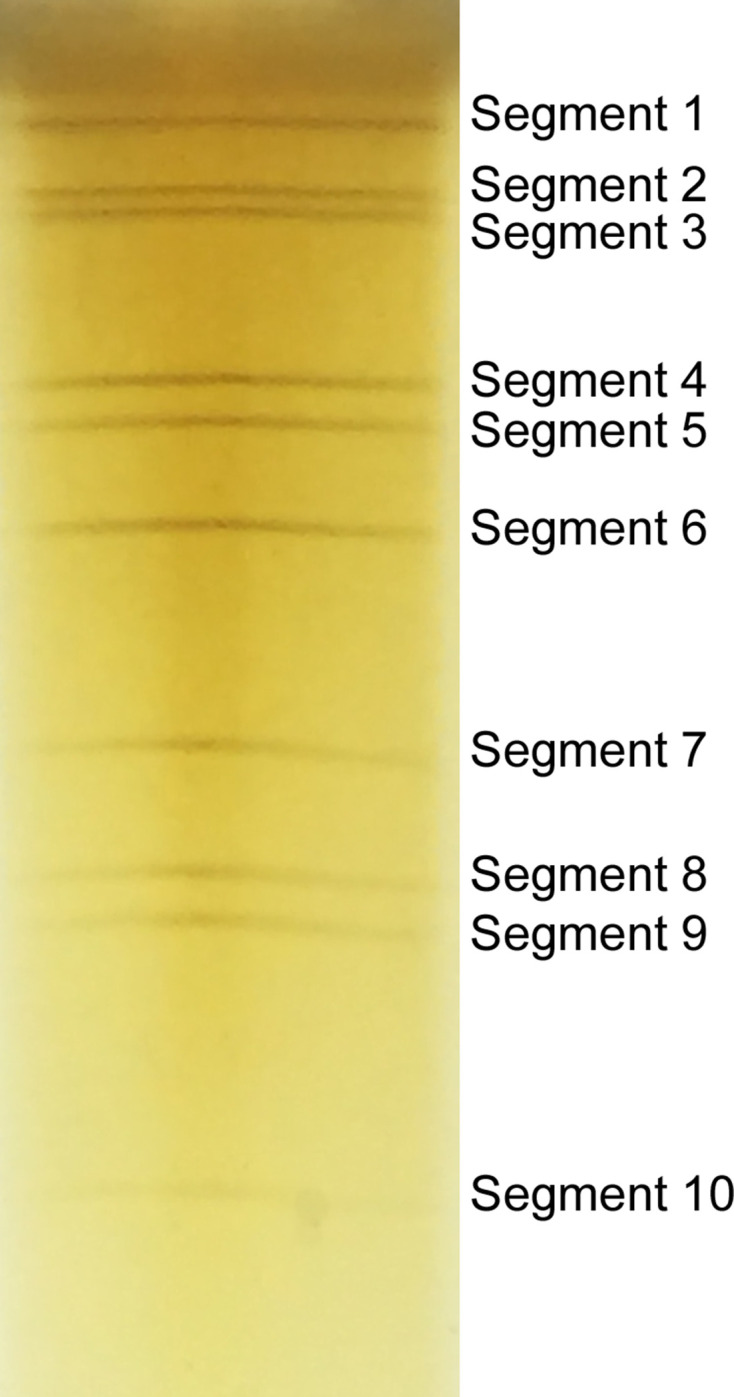
Electrophoresis profile of the dsRNAs of YN15-283-01 using a 10% acrylamide slab gel.

Viral RNA was harvested from the culture supernatant of infected BHK-21 cells and analysed by PAGE, revealing a ten-segment double-stranded RNA genome with a migration pattern of 3-3-3-1. Within this pattern, segment 10 was very weak but still identifiable (Fig. 2).

### Growth characteristics of the viral isolate in the cell cultures

To understand viral growth characteristics in a variety of host cells, the virus was inoculated into five mammalian cell lines, BHK-21, Vero E6, MDBK, SW13 and Huh 7, and two mosquito cell lines, C6/36 and Aag2, with different MOIs. This virus effectively replicated and caused a typical CPE characterized by cell exfoliation, shrinkage and death in BHK-21, Vero E6 and C6/36, however much weaker CPE was observed in SW13 cells. No CPE was observed in Aag2, Huh7 and MDBK cells (Figures S1–S6, available in the online version of this article).

**Fig. 3.**
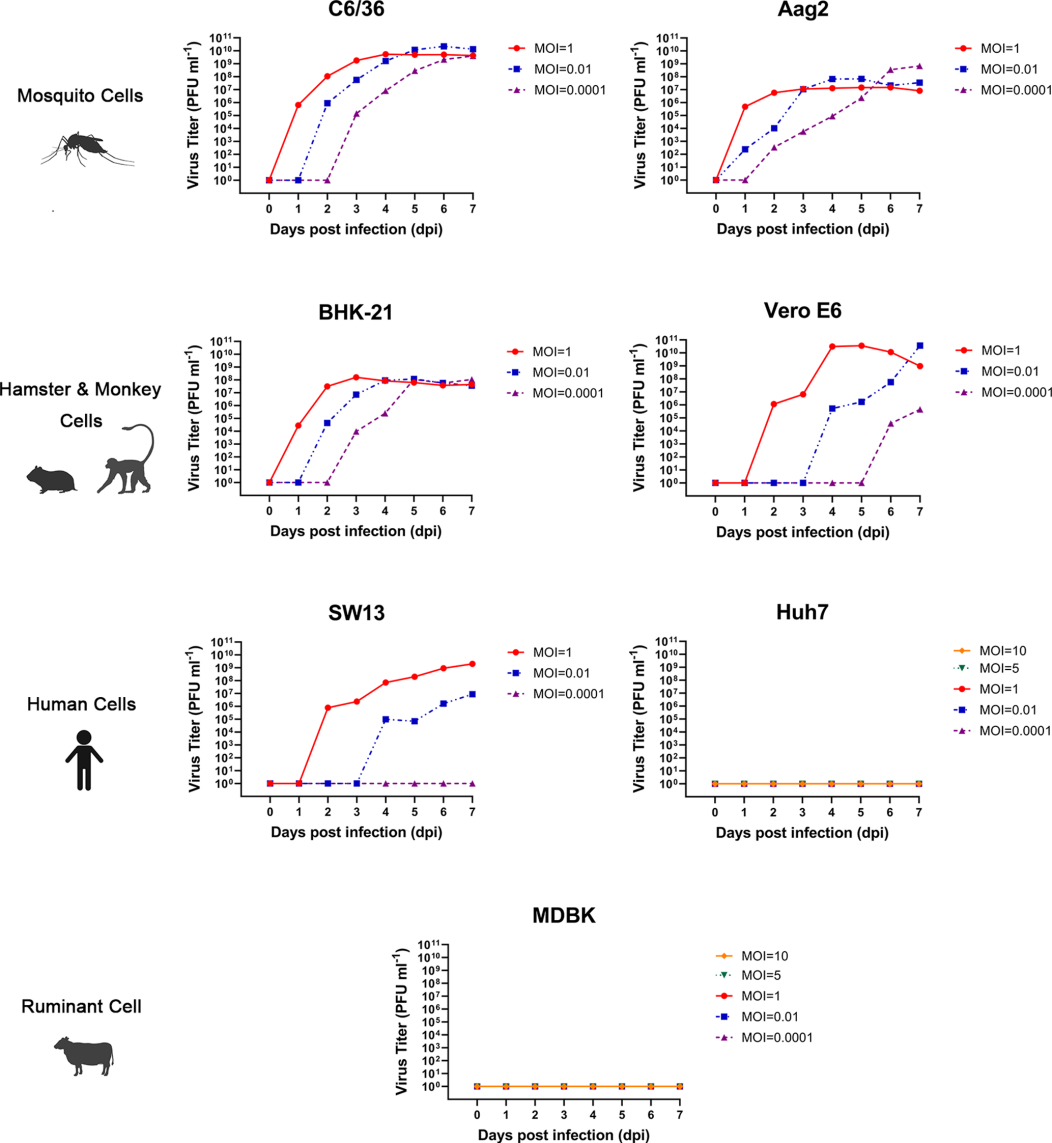
Growth curves of TIBOV YN15-283-01 with different MOIs in cells derived from mosquitoes and mammals. For C6/36, Aag2, BHK-21, Vero E6 and SW13 cells, the infected MOI=1, 0.01 and 0.0001; for Huh7 and MDBK cells, the infected MOI=10, 5, 1, 0.01 and 0.0001.

Viral growth kinetics analysis in the supernatant of the infected BHK-21 cells indicated that YN15-283-01 replicated efficiently and could reach peak titres of 10^8^ p.f.u. ml^−1^ and 10^9^ p.f.u. ml^−1^ when using MOIs of 1 and 0.01 or 0.0001, respectively, and then remained steady. Slight differences in Aag2 cells indicated that the virus titre peaked at 10^7^ p.f.u. ml^−1^ when usingMOIs of 1 or 0.01 but reached 10^9^ p.f.u. ml^−1^, with an MOI of 0.0001. The virus titres at an MOI of 1 reached the highest level on day four in Vero E6 cells and showed a downward trend, while the virus continued to multiply at MOIs of 0.01 and 0.0001. Viral replication was detected at an MOI of 1 or 0.01, but no viral growth was detected when using an MOI of 0.0001 in SW13 cells. In Huh7 and MDBK cell lines, the virus neither grew nor proliferated at either low or high MOIs from 0.0001 to 10. The results of statistical analysis indicated that there were no significant differences among the groups with the highest viral titres in different cell lines at the same MOI (MOI=1, 0.01 or 0.0001) (*P*>0.05). In addition, the differences were statistically significant in BHK-21 cells among different MOI virus treatments of the same cells (*P*<0.05), but there was no difference in other cell lines (*P*>0.05) (Fig. 3).

### Viral genome, phylogenetic and reassortment analysis of YN15-283-01

*De novo* assembly of the next-generation sequenced data acquired 99.8% coverage of the complete TIBOV genome. The lengths for segments 1–10 were 3950, 2901, 2769, 1978, 1775, 1640, 1165, 1142, 1103 and 833 bp, respectively. The complete CDSs of S1 to S10 encoded 1304 (VP1), 950 (VP2), 899 (VP3), 643 (VP4), 554 (NS1), 526 (VP5), 349 (VP7), 359 (NS2), 347 (VP6) and 234 (NS3) amino acids, respectively. The full genome sequences (S1 to S10) have been deposited in GenBank (accession numbers: MT793636 to MT79345) (Table 1).

Nucleotide sequence identity analysis of all 10 segments was conducted among YN15-283-01 and the other six TIBOV isolates with full genome information (Table 2). The results indicated that the genomic sequences of eight segments (S2 to S9) in YN15-283-01 share the highest identity (from 94.2–99.6 %) with the corresponding segments of SX-2017a isolated from *Culex tritaeniorhynchus* from Yunnan, whereas S1 and S10 of YN15-283-01 show the most similarity with XZ0906 acquired from *Anopheles maculatus* from Tibet, indicating that YN15-283-01 may have undergone a reassorting event between SX-2017a and XZ0906.

**Table 2.** Nucleotide sequence identity matrix for Tibet orbiviruses

Segments	S1	S2	S3	S4	S5	S6	S7	S8	S9	S10
Genes	VP1	VP2	VP3	VP4	NS1	VP5	VP7	NS2	VP6	NS3
TIBOV isolates	YN15-283-01									
**XZ0906**	0.974*	0.544	0.802	0.956	0.929	0.702	0.951	0.883	0.970	0.984*
**SX-2017a**	0.910	0.993*	0.942*	0.994*	0.996*	0.981*	0.996*	0.977*	0.984*	0.979
**DH13C120**	0.911	0.967	0.802	0.959	0.930	0.959	0.955	0.971	0.980	0.979
**D181/2008**	0.934	0.970	0.797	0.973	0.962	0.965	0.928	0.973	0.974	0.849
**KSB-8/C/09**	0.970	0.436	0.801	0.934	0.932	0.645	0.969	0.777	0.964	0.721
**KSB-3/C/10**	0.973	0.552	0.798	0.939	0.929	0.695	0.984	0.882	0.966	0.980

*Bold type indicates the highest sequence identity value.

**Fig. 4.**
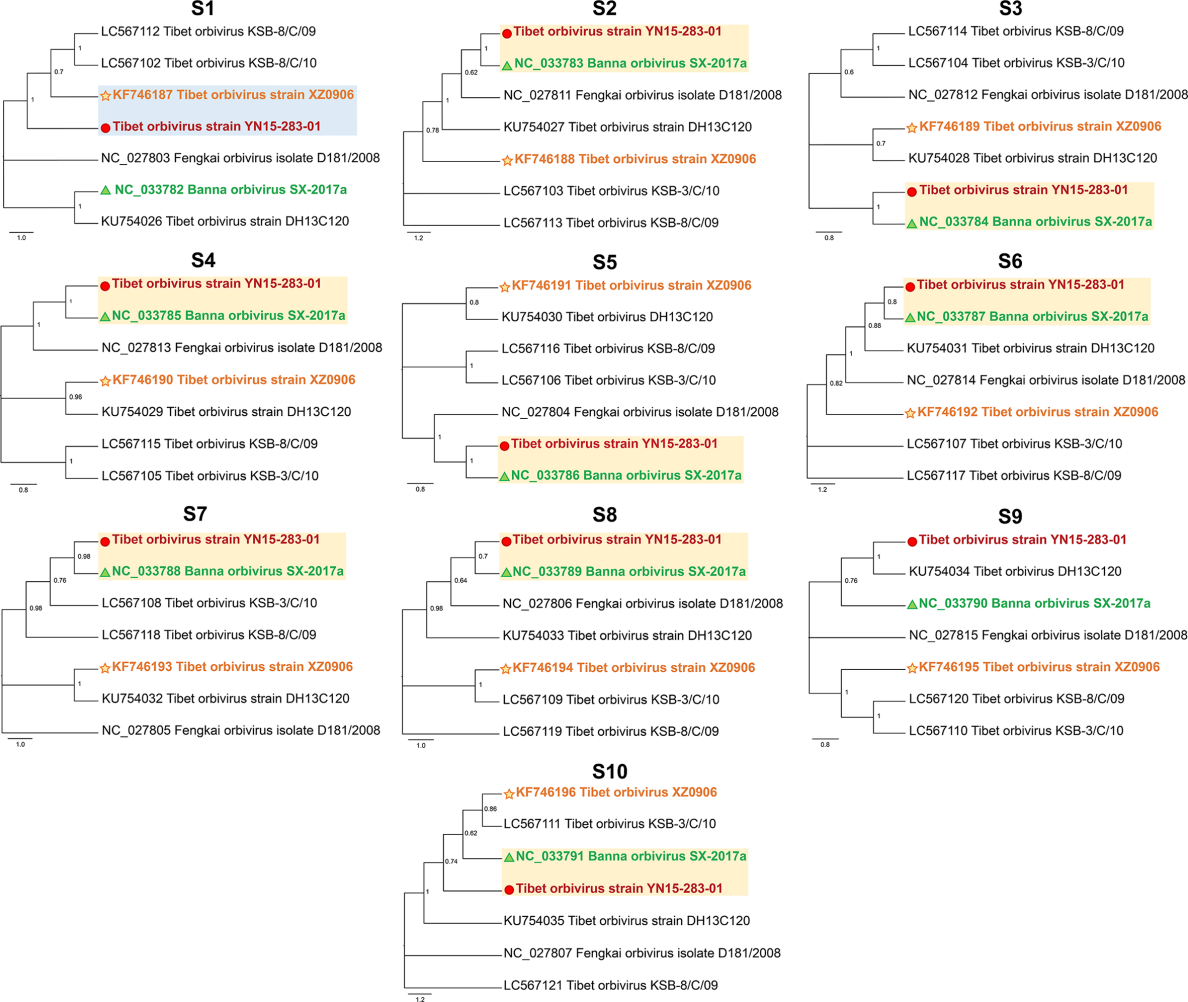
Maximum likelihood phylogenetic trees of S1–S10 segments for the TIBOVs and related orbiviruses. The scale bar indicates the evolutionary distance in the number of substitutions per nucleotide substitution/site, and the principal bootstrap support levels are indicated.

To further confirm the reassortment events, the phylogenetic tree was reconstructed based on each segment of all seven TIBOV strains. As shown in Fig. 4, the overall topology of the obtained phylogenetic trees with the S2 to S8 sequences were similar, as YN15-283-01 had the closest relationship with SX-2017a, while S1 of YN15-283-01 was clustered closely along with XZ0906.

**Fig. 5.**
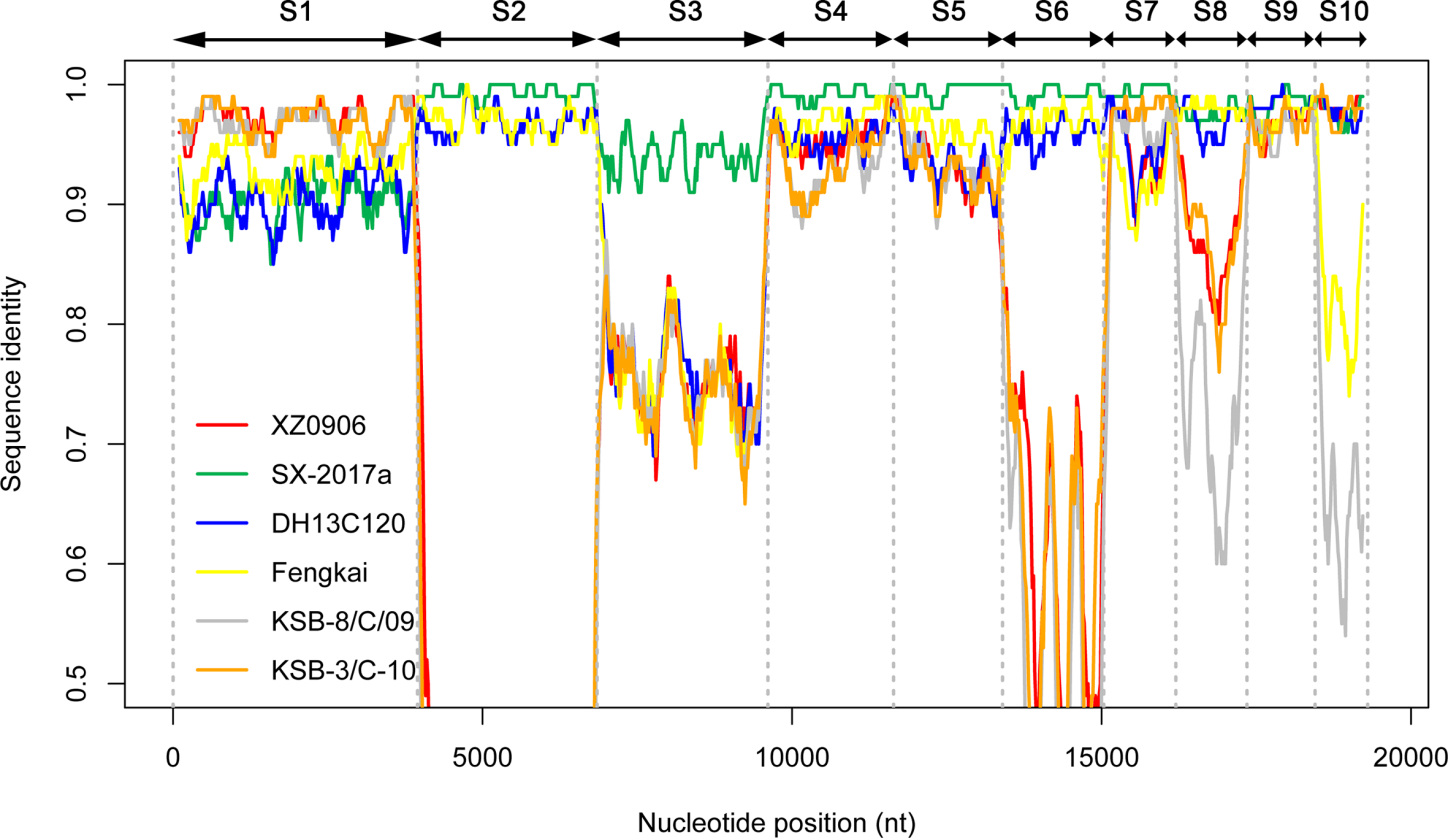
Simplot among seven TIBOV isolates. YN15-283-01 was used as the query, and the red, green, blue, yellow, grey and orange lines represent the strains XZ0906, SX-2017a, DH13C120, Fengkai, KSB-8/C/09 and KSB-3/C/10 respectively. The Y axis indicates the degree of sequence similarity, and the X axis indicates the position of genomic sequences of S1 to S10.

In addition, the Simplot (Fig. 5) and RDP4 analysis was used to detect the reassortment events in the genome of YN15-283-01, which were predicted using different algorithms (GENECONV, RDP, Bootscan, MaxChi, Chimaera, SiSscan and 3Seq) with a significance level set at *P*≤0.01. S1 of YN15-283-01 was potentially derived from XZ0906, S2, S4-S10 were derived from SX-2017a, whereas S3 was from an unknown source.

In summary, the results from similarity identity, phylogenetic and the Simplot/RDP4 reassortment analysis herein indicate that YN15-283-01 is a potential reassortment isolate that emerged from segment exchange mainly from XZ0906 and SX-2017a.

## Discussion

In this study, we isolated a Tibet orbivirus YN15-283-01 from *Culicoides* collected from Yunnan Province (PR China) in 2015. The genomic dsRNA electrophoresis profile indicated that the ten segments of YN15-283-01 were similar to those of other TIBOVs with 3-3-3-1 migration pattern. TYN15-283-01 can cause CPE in C6/36 cells, which is similar to the reported effects of strain YN12246 and Fengkai virus, but in contrast to strain XZ0906 which did not show CPE [34].

The genetic reassortment of viruses with 10 segments in the orbivirus plays an important role in driving the diversity and evolution of this virus group [13]. Herein, we performed reassortment analyses among all reported TIBOVs with full genome information, based on the results of phylogenetic, similarity identity and Simplot/RDP4 reassortment analysis. The results supported the hypothesis that the major parent of YN15-283-01 was SX-2017a, and the minor parent was XZ0906. The S1 of YN15-283-01 had the closest relationship with XZ0906, while S2, and S4–S8 were derived from SX-2017a. However, for the S3, even though in the phylogenetic tree YN15-283-01 was clustered with SX-2017a, the nucleotide identity between them was only 94.2%, and the RDP4 analysis indicated that the parent strains for S3 is unknown. This could be because of the limitations of the database for TIBOVs currently available, thus increased release of genomes in the future will help to improve the reassortment analyses.

Vector is the most important factor affecting arbovirus transmission and outbreaks. The midge feeding model has been developed to study infection, replication and dissemination of BTV in vectors [37]. Results from another study have indicated that *Culicoides sonorensis* are susceptible to EHDV [38, 39]. Although our results showed that YN15-283-01 can replicate efficiency in C6/36 and Aag2 cells, the experimental infection ofvectors, such as mosquito or midges, should be conducted in the future to determine the vector competence to the TIBOV.

It has been reported that intraperitoneal injection of Yunnan orbivirus into naïve mice led to productive, non-lethal viral replication and viremia [40], and the results of animal challenge experiments with strain DH13C120 of TIBOV indicated that it can lead to lethal neurovirulence in suckling mice and fever illness in sheep. In addition, the positive prevalence of DH13C120 virus and YN12246 strain antibodies has been detected in cattle and pigs in Yunnan Province [25]. These studies implied that TIBOV could be a potential pathogen leading to livestock diseases. More animal infection models should be developed to help understand the pathogenicity of TIBOV.

Presently, the general concept of One Health is widely accepted, but we do not know when and which disease will become the next outbreak, and multisectoral cooperation in the surveillance and control of emerging infectious diseases is quite challenging to achieve due to the significant gap between the fields of animal and human health [29]. However, systematic surveillance of vector-borne viruses is still essential and necessary to screen for vector-borne diseases that are underestimated and neglected [41]. Thus, it is important to study the host range, transmission and pathogenicity of TIBOV, then further assess the risks it may pose to public, veterinary or environmental health.

## Supplementary Data

Supplementary material 1
