# Supplementary material for: Characterization of a novel reassortment Tibet orbivirus isolated from Culicoides spp. in Yunnan, PR China
Source: J Gen Virol. 2021 Sep 8;102(9):001645. doi: 10.1099/jgv.0.001645 (PMC8567429; doi:10.1099/jgv.0.001645)
Supplement: Supplementary material 1 [file jgv-102-1645-s001.pdf]

Table S1: Different cell growth under MOI=1

	The day 1	The day 4	The day 7
BHK-21	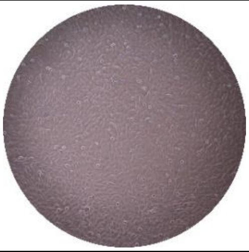	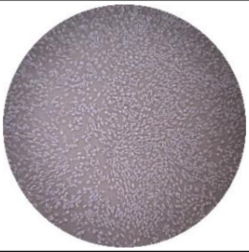	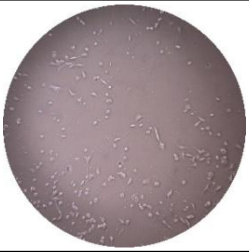
Vero-E6	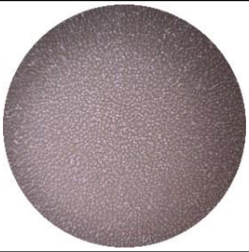	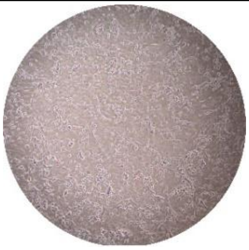	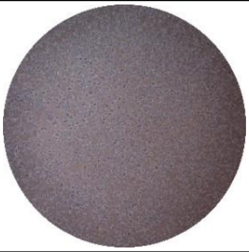
SW13	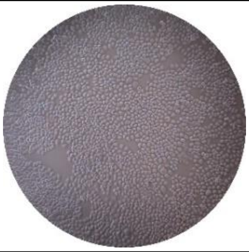	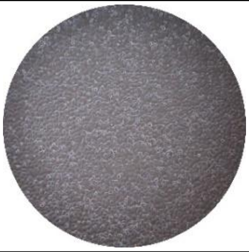	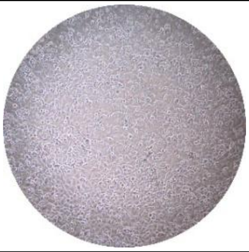
Huh 7	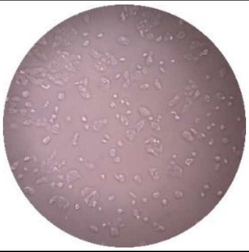	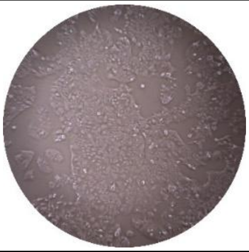	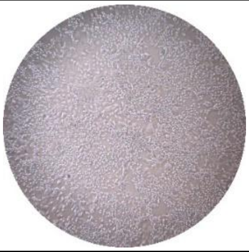
C6/36	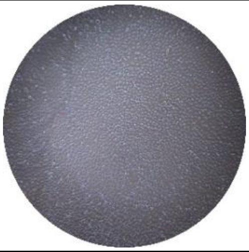	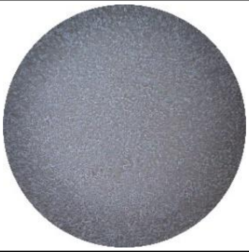	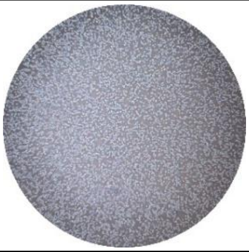
Aag 2	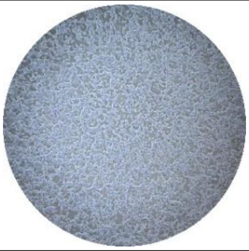	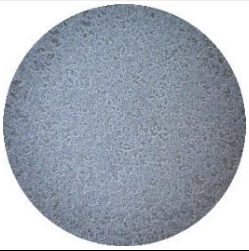	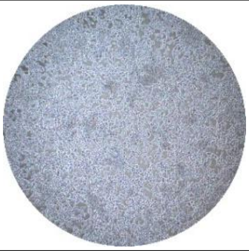
MDBK	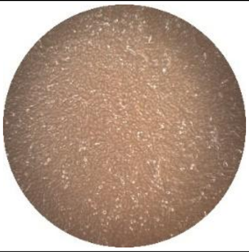	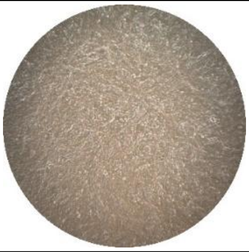	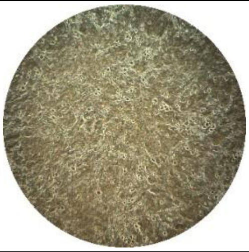

Table S2: Different cell growth under MOI=0.01

	The day 1	The day 4	The day 7
BHK-21	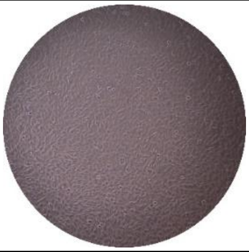	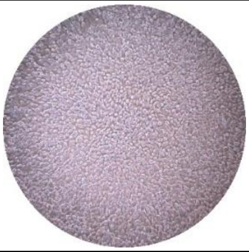	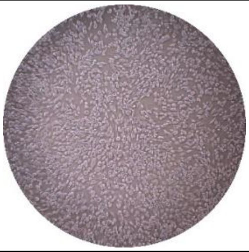
Vero-E6	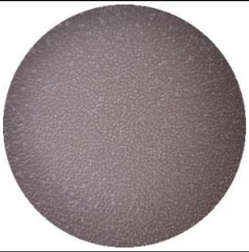	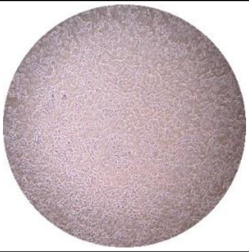	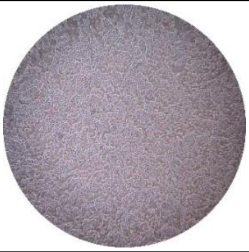
SW13	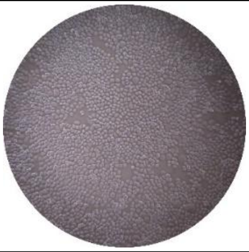	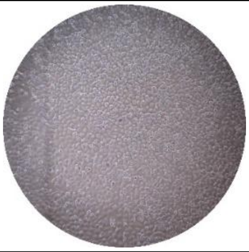	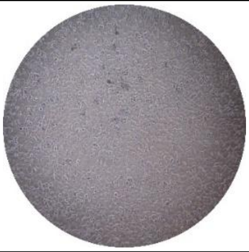
Huh 7	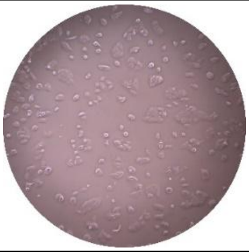	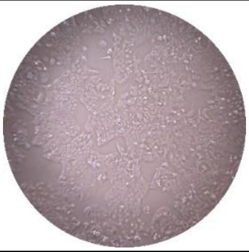	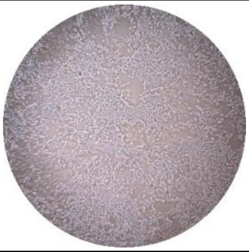
C6/36	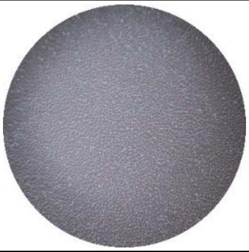	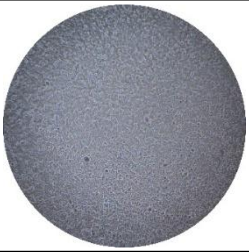	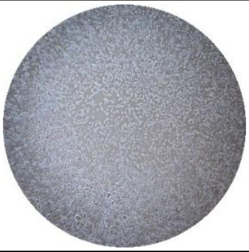
Aag 2	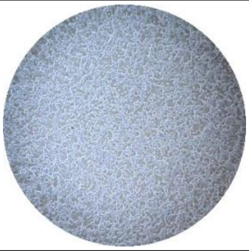	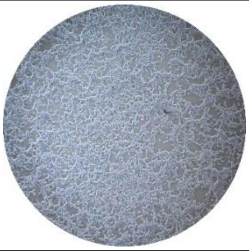	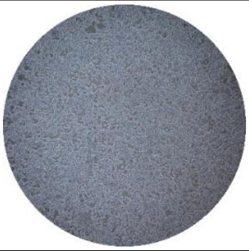
MDBK	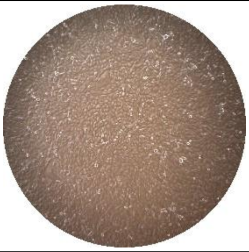	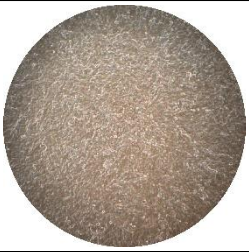	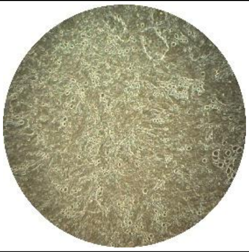

Table S3: Different cell growth under MOI=0.0001

	The day 1	The day 4	The day 7
BHK-21	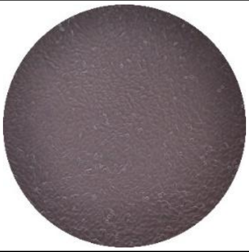	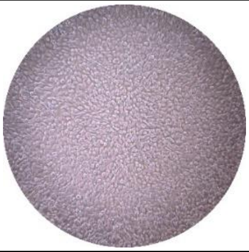	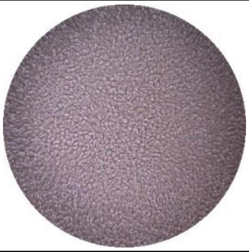
Vero-E6	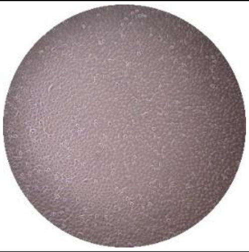	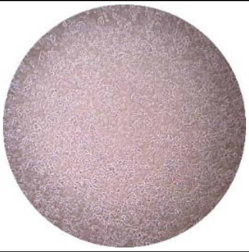	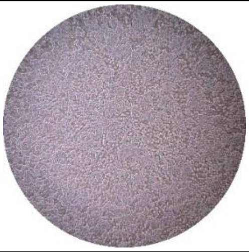
SW13	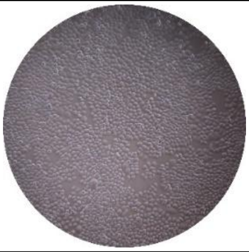	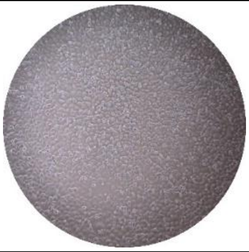	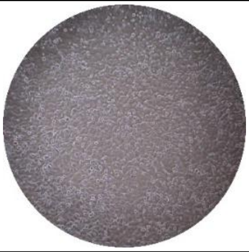
Huh 7	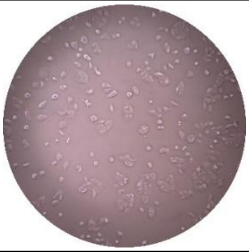	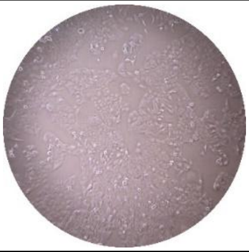	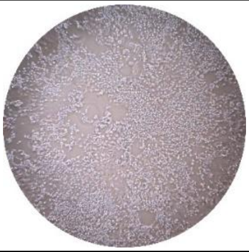
C6/36	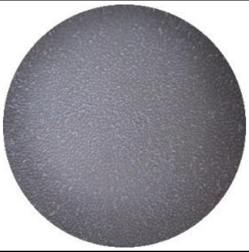	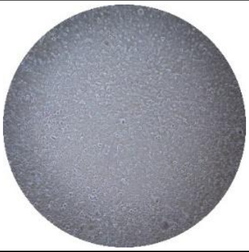	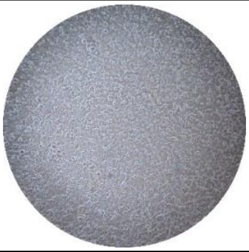
Aag 2	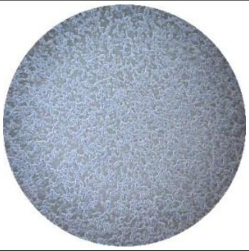	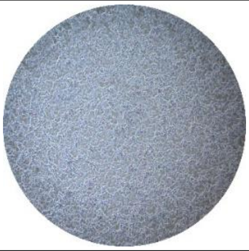	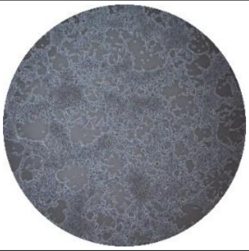
MDBK	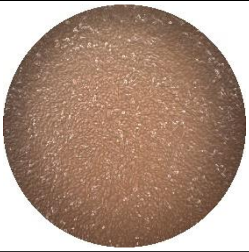	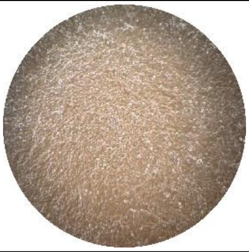	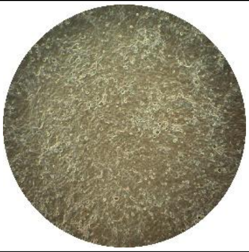

Table S4: Different cell growth as MOCK

	The day 1	The day 4	The day 7
BHK-21	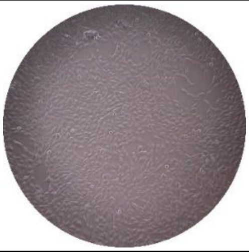	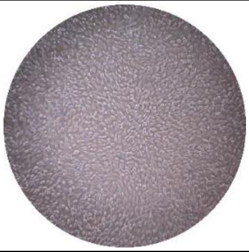	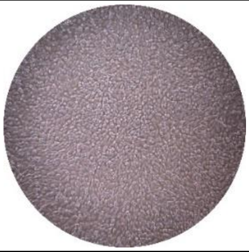
Vero-E6	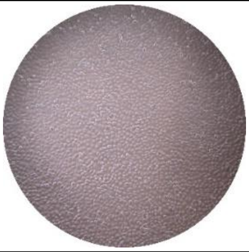	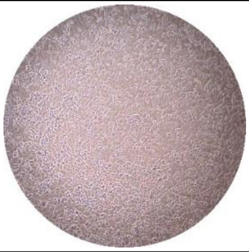	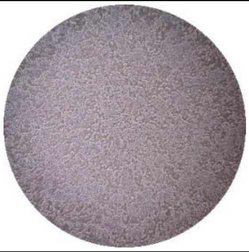
SW13	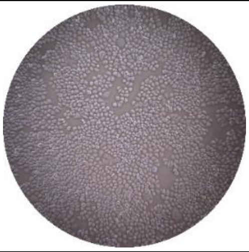	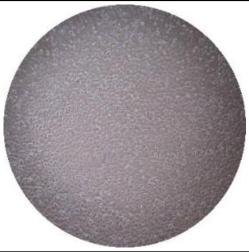	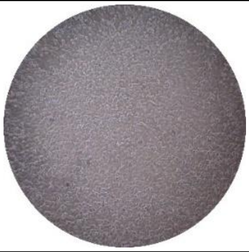
Huh 7	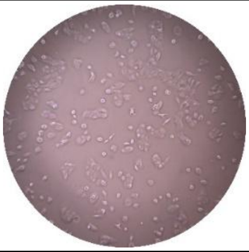	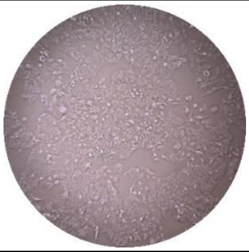	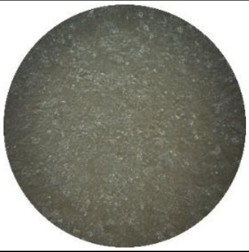
C6/36	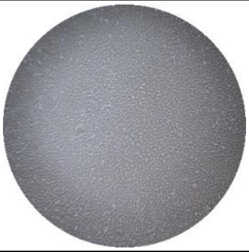	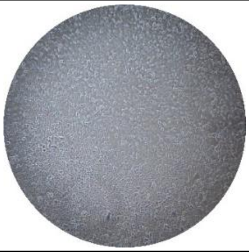	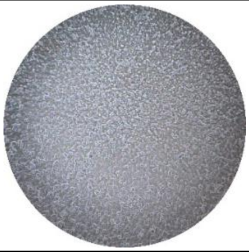
Aag 2	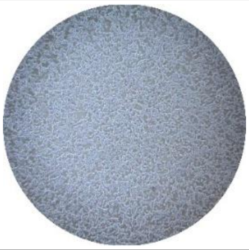	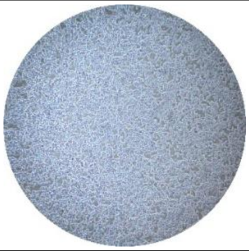	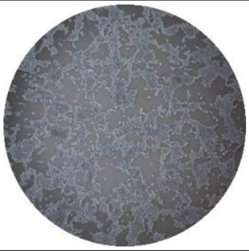
MDBK	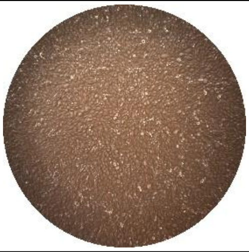	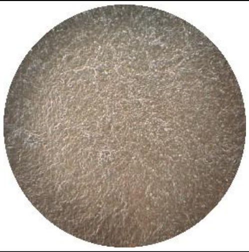	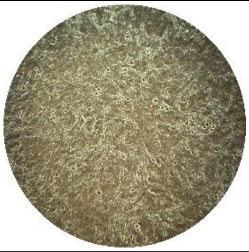

Table S5: Different cell growth under MOI=5

	The day 1	The day 4	The day 7
Huh 7	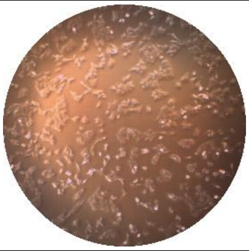	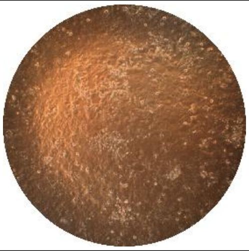	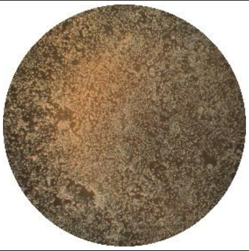
MDBK	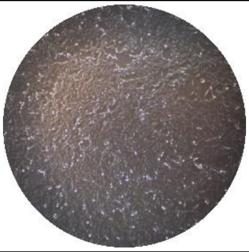	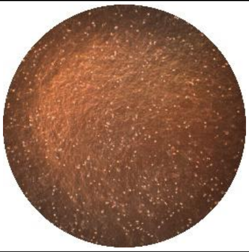	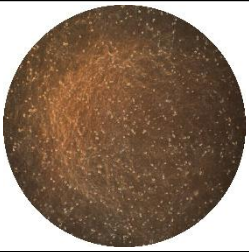

Table S6: Different cell growth under MOI=10

	The day 1	The day 4	The day 7
Huh 7	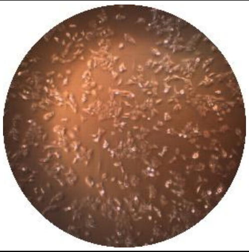	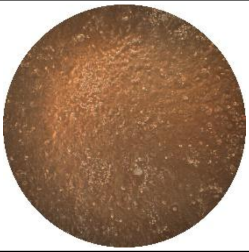	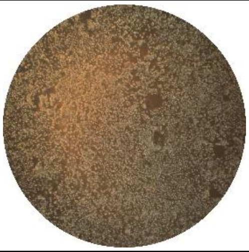
MDBK	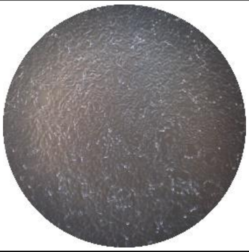	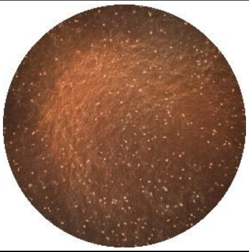	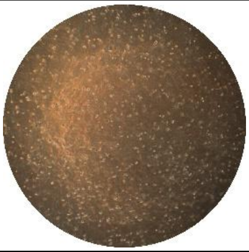
